# No effect of thyroid hormones on 5‐year mortality in patients with subjective cognitive decline, mild cognitive disorder, and Alzheimer’s disease

**DOI:** 10.1111/jne.13107

**Published:** 2022-02-25

**Authors:** Blaž Đapić, Eva Schernhammer, Helmuth Haslacher, Elisabeth Stögmann, Johann Lehrner

**Affiliations:** ^1^ 27271 Department of Neurology Medical University of Vienna Vienna Austria; ^2^ 27271 Department of Epidemiology Center for Public Health Medical University of Vienna Vienna Austria; ^3^ 27271 Department of Laboratory Medicine Medical University of Vienna Vienna Austria

**Keywords:** 5‐year mortality, Alzheimer’s disease, MCI, neuropsychological battery, SCD, thyroid hormones

## Abstract

The present study aimed to investigate differences in circulating thyroid hormone levels, gender, education, depressive symptoms, and cognitive performance among patients with cognitive impairment, and also to examine their associations, as well as that of cognitive decline, with 5‐year mortality. Between 1998 and 2017, a hospital‐based, single‐centre (Neurology Department of the General Hospital in Vienna, Austria), retrospective follow‐up study enrolled 2102 patients with mild to severe cognitive impairment (grouped into subjective cognitive decline, mild cognitive impairment, and Alzheimer's disease). Cox proportional hazards models were used to calculate hazard ratios (HRs), with 95% confidence intervals (CIs), as well as to calculate stepwise adjustments for demographic variables (age, gender, and education), depressive symptoms (Geriatric Depression Scale, GDS‐15), and neuropsychological abilities (four domains of a neuropsychological test battery of Vienna, NTVB). In univariate analyses, total triiodothyronine (TT3) levels differed significantly between Alzheimer's disease and mild cognitive impairment patients (*p*_diff_ = .001). No other differences in cognitive impairment subgroups with any of the measured thyroid hormones were observed. Furthermore, in multivariate models, circulating TT3 was not associated with mortality (multivariable‐adjusted HR per unit increase in TT3 = 0.56; 95% CI = 0.29–1.07). In multivariate models, we observed significantly lower 5‐year mortality among women (HR = 0.56; 95% CI = 0.43–0.73) and those who scored higher on any of the four domains of the NBTV (e.g., attention and perceptual speed, HR = 0.63; 95% CI = 0.54–0.72); we also observed significantly higher 5‐year mortality among patients with depressive symptoms (HR per one point score increase in GDS‐15 = 1.06; 95% CI = 1.02–1.10), regardless of cognitive impairment subgroup. Among patients with various degrees of cognitive impairment, we found no associations of thyroid hormone levels with 5‐year mortality. Gender, neuropsychological abilities, and depressive symptoms were each significant predictors of 5‐year mortality. These results suggest that a neurocognitive test performance could serve as an important predictor of 5‐year mortality among patients with cognitive impairment, although further studies with a more complete adjustment for comorbidities are needed to confirm these findings.

## Abstract 2

Among patients with various degrees of cognitive impairment, we found little to no differences in circulating thyroid hormone levels and no associations of thyroid hormone levels with mortality. Further studies with more complete adjustment for comorbidities are needed to confirm these findings.

## 1 INTRODUCTION

Dementia is one of the most common causes of death in the senior population,^1^, ^2^, ^3^ with Alzheimer's disease (AD) representing more than half of all diagnosed senile dementias.^4^ However, cognitive decline does not always happen rapidly and there are transitional stages. Subjective cognitive decline (SCD) as the earliest diagnostic stage of dementia on the trajectory of cognitive decline is identified as perceived cognitive decline, in which only the afflicted person notices differences of present cognitive abilities compared to the previous ones.^5^ Patients with more serious mild cognitive impairment (MCI)^6^ (i.e., the next stage on the dementia trajectory) exhibit a higher risk of developing Alzheimer's disease (i.e., the final stage on the trajectory to dementia), as well as other forms of cognitive impairment.^7^ Previous studies have consistently shown higher mortality rates in senior populations with MCI or AD.^2^ Early symptoms of AD are mostly pronounced as episodic memory problems, followed by difficulties in executing a complex task, followed by the inability to perform everyday activities. Behavioral changes set in, followed by disorientation, confusion, impaired mobility, and potential hallucinations.^8^ Ultimately, motoric difficulties lead to swallowing and malnutrition, which leads to the acquisition of often fatal pneumonia among the already weak patients.^9^

Additional factors such as age, gender, and comorbidities, including cardiovascular diseases and diabetes mellitus, influence the increased mortality of patients with dementia, regardless of the severity of their cognitive decline,^10^ although cognitive decline in itself as measured by various cognitive test batteries has also been associated with an increased mortality in the geriatric population.^11^ Furthermore, depression is also an important factor because Byers et al.^12^ found that veterans with depression or dysthymia have a higher change of developing dementia and have a higher mortality rate than veterans in an euthymic state. Another factor of interest comprises the circulating thyroid hormones.

Thyroid dysfunctions are very common in the general population and are associated with an increased risk of cardiovascular diseases, diabetes, various cancers, lung diseases, and psychiatric diseases.^13^ Women and the elderly are most at risk of having thyroid dysfunction.^14^ Similar to most organs in our body, the thyroid gland is affected by the passing of time, which can be beneficial or disadvantageous for overall survival. Thyroid‐stimulating hormone (TSH) levels generally rise in older adults, and a subclinical hypothyroid state may be beneficial to longevity as reported by Lang et al.^14^ Gusseklo et al.^15^ showed that unusually high levels of TSH and low levels of free thyroxin (fT4) might be associated with a low mortality rate among the senior population compared to older patients with low TSH levels and high fT4 levels, who had the highest mortality rate. Another study^16^ found that older men between the ages of 73 and 94 years with lower free fT4 serum levels had a higher 4‐year survival rate than older men with normal fT4 serum levels. By contrast, Laulund et al.^13^ reported that thyroid dysfunctions, such as overt and subclinical hyperthyroidism, as well as overt hypothyroidism, have a significant association with an increased mortality rate.

In light of this mixed evidence for thyroid hormones, we hypothesized that there is a difference in circulating thyroid hormones between SCD, MCI, and AD patient groups, as well as for demographic factors (gender, education), depressive symptoms, and cognitive performance. We also hypothesized that the above mentioned factors and cognitive decline will have an effect on the 5‐year mortality of patients affected with SCD, MCI, and AD.

## 2 MATERIALS AND METHODS

The present study represents a retrospective, single‐centre analysis, with all data having been collected over a period of almost 20 years (January 1998 to December 2017) from patients within the Neurology Department of the Medical University of Vienna. Of note, subjects with SCD were only documented starting in 1999. The relevant data for the present study were extracted from the Research, Documentation, and Analysis (RDA) system of the Medical University of Vienna, or the Allgemeines Krankenhaus Information's Management system (AKIM) in a corresponding SPSS (IBM Corp.) data matrix, and subsequently analyzed. If no death certificate was confirmed until 31 December 2017 in either the RDA or AKIM, then the patient was assumed to be alive.

### 2.1 Participants

Patients were diagnosed and referred by physicians from the Department of Neurology or they had a self‐complaint about memory problems in recent times. Patients were diagnosed on the basis of their cognitive performance into three diagnostic groups: SCD, MCI, or AD. All participants had a neurological examination, using criteria from Jessen et al.^5^ for the diagnosis of SCD, and Petersen's criteria from 2004 for the diagnosis of MCI.^17^ NINCDS‐ADRDA guidelines were taken into account for the diagnosis of AD.^18^, ^19^ Serum thyroid hormone levels were measured in blood samples collected from the patients at one of their visits to the Department of Neurology. Study participants were included in the study if they were at least 50 years of age at the time of neuropsychological testing. People who had a stroke, a serious head injury or psychiatric problems causing pseudo‐dementia were excluded from the study. The subjects were given the Mini‐Mental State Examination (MMSE),^20^ the Geriatric Depression Scale (GDS‐15),^21^ and the Neuropsychological Test Battery Vienna (NTBV‐short).^22^ The study was approved by the ethics committee of the Medical University of Vienna and followed the tenants of the Declaration of Helsinki.

Information about circulating thyroid levels was taken from the AKIM‐System. Only values that were acquired ±3 months within the neuropsychological setting were taken into account. Some patients had incomplete blood parameters, with one or more missing thyroid parameters. If patients did not have a TSH value, or the existing thyroid parameters did not match the neuropsychological test date within a ±3‐month period, they were excluded from the analyses.

**FIGURE 1:**
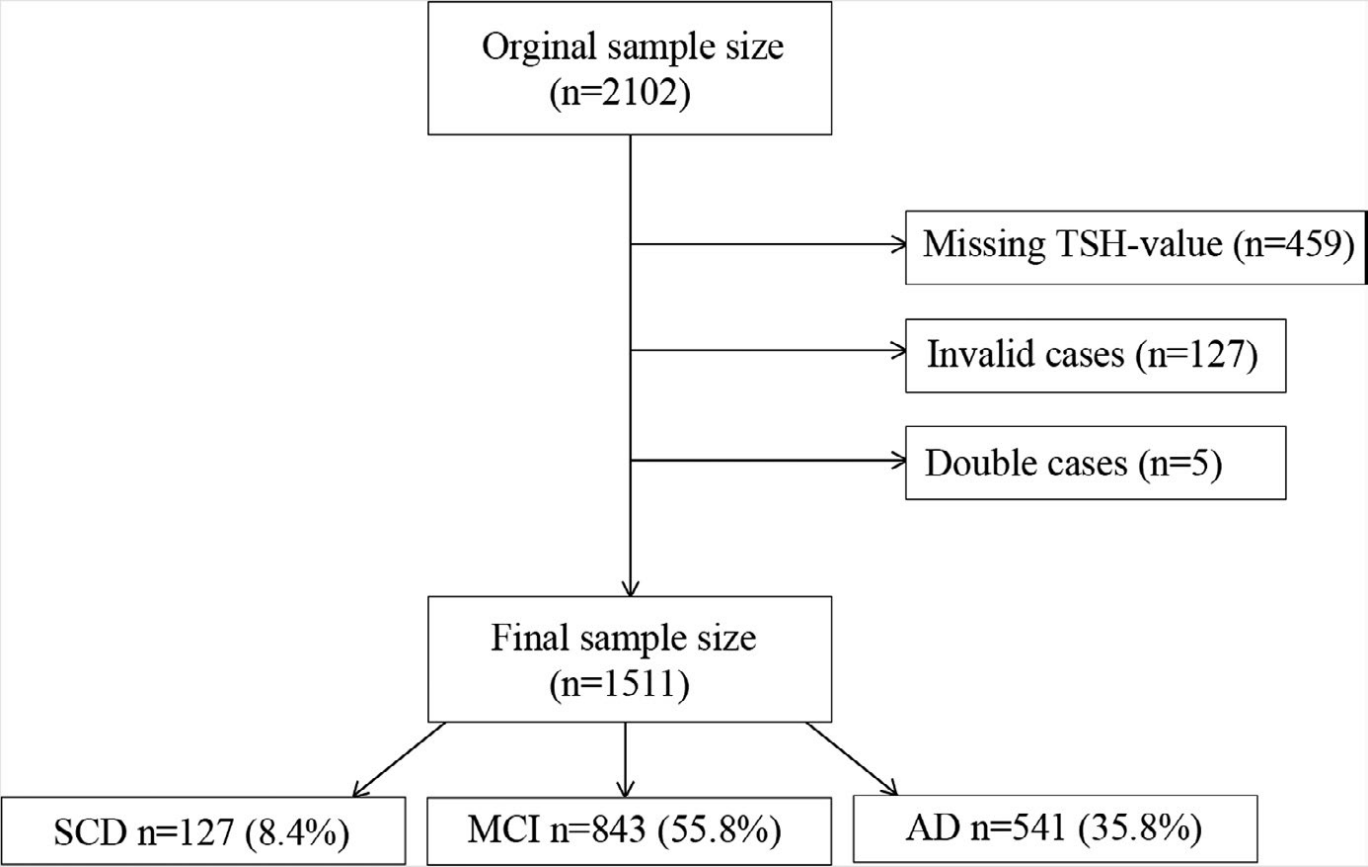
Flowchart, showing the exclusion of cases from the study and the remaining participants. AD, Alzheimer's disease; MCI, mild cognitive impairment; SCD, subjective cognitive decline; TSH, thyroid‐stimulating hormone

Of the 2102 individual patients whose data were retrieved at the beginning, 591 did not meet the required criteria of the study. Specifically, 459 individuals lacked thyroid parameters (specifically TSH parameters) and 127 had invalid data or were too young to participate in the study. An additional five patients had to be removed as a result of double counting, leaving a total of 1511 patients as our base population. Figure 1 illustrates the exclusion protocol for patients who did not meet the required criteria of the present study.

### 2.2 Instruments

#### 2.2.1 Thyroid parameters

Serum TSH, total thyroxin (TT4), fT4, total triiodothyronine (TT3), and free triiodothyronine (fT3) levels were implemented using standard laboratory values used in General Hospital Vienna (AKH‐Wien) using the electrochemiluminescence immunoassay method. Normal ranges for thyroid hormones are: 0.27–4.2 µIU mL^–1^ (TSH), 0.8–1.8 ng mL^–1^ (TT3), 58–124 ng mL^–1^ (TT4), 2.15–4.12 pg dL^–1^ (fT3), and 0.76–1.66 ng dL^–1^ (fT4).

#### 2.2.2 Neuropsychological instruments

Neuropsychological tests were used to determine and evaluate the cognitive performance among the subjects in the study. MMSE score was not included in descriptive statistics or further analyses because of its redundant role as a neuropsychological test in the place of the NTBV‐15, a modified short version of NTBV that is also used to determine Parkinson's disease.^22^ Furthermore, NTBV‐short was included, which measures categories such as psychomotor speed, concentration or attention, language, memory, or executive functions.^22^ Finally, the GDS‐15 was included, which is a questionnaire with yes/no answers designed to measure geriatric depression.^21^

### 2.3 Statistical analysis

For descriptive purposes, we calculated the mean ± SD, and present the range of minimum (min) and maximum (max). If the distribution of data was skewed, we present medians and interquartile ranges (IQR) (25%–75%). To deduce whether the frequency of distribution was skewed, Kolmogorov–Smirnov and Shapiro–Wilk tests were used.

Additionally, analyses were performed utilizing the non‐parametric Kruskal–Wall test and the Mann–Whitney *U* test to compare the diagnostic subgroups.

Cox proportional hazards models were used to examine associations of thyroid parameters and varying levels of cognitive impairment (SCD, MCI, and AD) with mortality. Because, in univariate analyses, the results of Kruskal–Wallis testing revealed significant differences by diagnostic subgroups only for TT3 (χ^2 ^= 11.332, *df* = 2, *p* = .003), and all other thyroid parameters (TSH, fT3, fT4, and TT4) showed no significant difference between diagnostic subgroups (*p* ≥ .168), only TT3 was retained for multivariable testing.

Patients were followed until they were deceased or until the end of follow‐up (31 December 2017), whichever came first. Multivariable models adjusted for age, gender, and number of years in school (representative of educational status), NTVB‐short (representing neurocognitive functioning), and GDS‐15 score (representing depressive symptoms). The calculation was performed using five hierarchical blocks via the enter method, where, in each block, the predictors were inserted simultaneously. All statistical analyses were performed using SPSS, version 26 (IBM Corp.). *p* < .05 (two‐sided) was considered statistically significant. Bonferroni adjustment was performed to account for multiple testing.

## 3 RESULTS

Demographics, clinical and circulating thyroid parameters data are summarized in Table 1.

**TABLE 1:** Characteristic values (mean, SD, median, interquartile ranges, and frequency [%] and proportion) of observed data according to the diagnostic subgroups

	Diagnostic subgroup			
	SCD	MCI	AD	Total
Age (years) at entry into the study	*n* = 127	*n* = 843	*n* = 541	*N* = 1511
	65.5 (59.8; 74.5)	69.7 (61.5; 75.9)	76.0 (70.2; 80.8)	71.9 (63.7; 78.0)
Age (years) of death	*n* = 17	*n* = 220	*n* = 291	*N* = 528
	85.8 (72.8; 89.50)	82.6 (75.6; 88.0)	82.4 (77.1; 87.0)	82.6 (76.6; 87.3)
Gender (male)	60 (47.2%)	365 (43.3%)	233 (43.1%)	658 (43.5%)
Gender (female)	67 (52.8%)	478 (56.7%)	308 (56.9%)	853 (56.5%)
School years	*n* = 127	*n* = 843	*n* = 541	*N* = 1511
	12.0 (8.0; 15.0)	11.0 (8.0; 15.0)	8.0 (8.0; 12.0)	10.0 (8.0; 14.0)
GDS‐15 score (0–15)	*n* = 121	*n* = 729	*n* = 341	*N* = 1191
	3.0 (1.0; 5.0)	3.0 (2.0; 6.0)	3.0 (2.0; 5.5)	3.0 (2.0; 6.0)
NTBV factor	*n* = 124	*n* = 733	*n* = 258	*N* = 1142
F1 attention and perceptual speed	0.561 (0.125; 0.929)	0.225 (−0.319; 0.744)	−0.461 (−1.488; 0.268)	0.132 (−0.466; 0.690)
F2 verbal memory	0.700 (0.215; 1.053)	0.264 (−0.316; 0.825)	−0.827 (−1.516; −0.162)	0.087 (−0.658; 0.734)
F3 executive functions	0.240 (−0.087; 0.852)	0.148 (−0.463; 0.732)	−0.379 (−1.087; 0.345)	0.067 (−0.599; 0.676)
F4 verbal fluency and semantic memory	0.532 (0.032; 1.081)	0.052 (−0.530; 0.716)	−0.361 (−1.191; 0.345)	0.002 (−0.605; 0.692)
TSH (µIU mL^–1^)	*n* = 127	*n* = 843	*n* = 541	*N* = 1511
	1.623 ± 0.990	1.799 ± 1.751	1.834 ± 3.238	1.797 ± 2.354
TT3 (ng mL^–1^)	*n* = 113	*n* = 734	*n* = 456	*n* = 1303
	1.04 (0.94; 1.16)	1.07 (0.93; 1.19)	1.02 (0.91; 1.15)	1.04 (0.92; 1.17)
TT4 (ng mL^–1^)	*n* = 112	*n* = 736	*n* = 457	*n* = 1305
	76.07 ± 13.65	76.52 ± 16.03	77.36 ± 14.84	76.78 ± 15.43
fT3 (pg dL^–1^)	*n* = 8	*n* = 48	*n* = 54	*n* = 110
	2.999 ± 0.308	2.848 ± 0.807	2.705 ± 0.533	2.789 ± 0.658
fT4 (ng dL^–1^)	*n* = 16	*n* = 74	*n* = 69	*n* = 159
	1.216 ± 0.211	1.256 ± 240	1.250 ± 0.237	1.250 ± 0.235

Abbreviations: AD, Alzheimer's disease; MCI, mild cognitive impairment; SCD, subjective cognitive decline; GDS, Geriatric Depression Scale; NTBV, Neuropsychological Test Battery Vienna; TSH, thyroid‐stimulating hormone; TT3, triiodothyronine; TT4, thyroxin; fT3, free triiodothyronine; fT4, free thyroxin.

### 3.1 Circulating thyroid parameters

The results of Kruskal–Wallis testing revealed significant differences in TT3 (χ^2^ = 11.332, *df* = 2, *p* = .003. Other thyroid parameters (TSH, fT3, fT4, and TT4) showed no significant difference between diagnostic subgroups (*p* ≥ .168). Pairwise comparison post‐hoc performing *U* tests showed significantly higher values, with a weak effect only for MCI vs. AD concerning TT3 (*U* = 148044.0, *z* = −3.350, *p* = .001, *r* = .10).

These results compelled us to only use the TT3‐value as a representative of the thyroid parameters because other parameters revealed no significant differences among the cognitive diagnosis subgroups. Accordingly, TT3 was the only thyroid parameter used in the subsequent Cox proportional hazards model to analyze the impact on the 5‐year mortality of patients with cognitive degeneration.

### 3.2 Neuropsychological functions and depressive symptoms

NTBV‐15 short subtest results were first analyzed utilizing a principal component analysis, using the varimax rotation method with Kaiser normalization to achieve a dimensional reduction. The rotation converged in nine iterations, and a four‐dimensional solution can be suggested, which represents the information contained in an optimized way, as shown in Table 2. The information shows a Kaiser–Meyer–Olkin test statistic (KMO = 0.891) with sufficient extent of information for conducting an explorative factor analysis. The explained variance proportion achieved 76.1% for all extracted components together, representing *n* = 1142 cases with complete data protocols.

**TABLE 2:** Rotated component matrix‐factor loadings, the communality of items and eigenvalue of factors (*n* = 1142)

NTBV−15 short subtest	Factor				Communality
	1	2	3	4	$h_{i}^{2}$
Concentration I	−0.810	−0.226	−0.228	−0.086	0.77
Interference time (c.I.)	−0.793	−0.203	−0.175	−0.290	0.79
Concentration II	0.761	0.282	0.253	0.141	0.74
Interference total/time (c.I.)	0.760	0.223	0.150	0.304	0.74
Symbols (c.I.)	−0.754	−0.197	−0.174	−0.149	0.66
Psychomotor processing speed	−0.663	−0.278	−0.370	−0.164	0.68
VSRT delayed retrieval	0.238	0.861	0.158	0.177	0.85
VSRT learning performance	0.336	0.849	0.140	0.219	0.90
VSRT word span	0.283	0.806	0.090	0.161	0.76
VSRT recognition	0.117	0.779	0.168	0.074	0.66
Labyrinth time	−0.497	−0.195	−0.758	−0.091	0.87
Labyrinth total/time	0.432	0.203	0.756	0.077	0.81
PWT‐f correct	0.381	0.168	−0.017	0.758	0.75
Confrontation naming (BNT)	0.034	0.222	0.547	0.617	0.73
Animal naming (word fluency)	0.428	0.414	0.180	0.576	0.71
Eigenvalue (λ)	4.48	3.38	1.90	1.66	11.42
Explained variance component	29.8%	22.6%	12.6%	11.1%	76.1%

Abbreviations: BNT, Boston Naming Test; NTBV, Neuropsychological Test Battery Vienna; PWT, Phonematic Verbal Fluency Test; VSRT, Verbal Selective Reminding Test.

The naming of the uncorrelated factors combined into four categories was carried out with the following key terms: (1) *Attention and perceptual speed*, (2) *Verbal memory*, (3) *Executive functions*, and (4) *Verbal fluency and semantic memory*.

Subsequently, NTBV factor *z*‐score performances were compared between diagnostic subgroups using Kruskal–Wallis testing. The test value *H* of the Kruskal–Wallis test showed a significant difference in factor scores between the diagnostic subgroups in each case (*p* < .001). Likewise, with post‐hoc pairwise comparisons using the *U* test procedure between diagnostic subgroups, the Mann–Whitney *U* test also showed a significant difference between SCD vs. AD, SCD vs. MCI, and MCI vs. AD for all four‐factor scores (*p* ≤ .008) (Bonferroni correction, α* = 0.0167). The results indicate a hierarchical ranking of cognitive performance: SCD > MCI > AD. These four‐factor scores were included as 5‐year mortality predictors in the following Cox proportional hazards model.

The results of Kruskal–Wallis testing revealed significant differences in GDS level, χ^2^ = 6.776, *df* = 2, *p* = .034. Pairwise comparison post‐hoc *U* tests showed significantly higher values for MCI compared to SCD, indicating a weak effect (*U* = 37883.5, *z* = −2.502, *p* = .012, *r* = .09), especially when taking Bonferroni adjustment into account (α* = .0167). The remaining pairwise comparisons concerning GDS‐15 did not show any further significant differences for the subgroup comparisons.

### 3.3 Model for influencing factors on 5‐year mortality

To summarize all the previous results, we used a Cox proportional hazards model. In the model, 61.6% of the patients, in total 931 participants (*n*_SCD_ = 106, *n*_MCI_ = 612, *n*_AD_ = 213), could be included. Of these, 245 (26.3%) were deceased, whereas 686 (73.7%) were censored cases. This multiple regression procedure started in blockwise order with the inclusion of TT3 (ng mL^–1^), representative for thyroid functioning. Then, cognitive diagnosis groups (SCD, MCI, AD), gender, school years (representing education), and GDS‐15 scores (representing depressive symptoms) were added. Finally, in the fifth block, the four‐factor scores with NTVB‐15 short abilities (representing neuropsychological functioning) were included.

In the first block of the Cox regression, only the TT3 thyroid parameters (ng mL^–1^) were analyzed. The result was significant, showing that patients with lower circulating TT3 levels had a higher risk of dying sooner (*p* < .001).

In the second block of the model, we included cognitive diagnostic groups. The result was significant, showing that patients with a more severe diagnosis had a higher 5‐year mortality risk (*p* ≤ .013). TT3 levels remained significant (*p* = .005), meaning that lower TT3 levels are still a risk factor.

In the third block, we included the sociodemographic parameters of school years and gender. In the case of gender, men tended to die sooner than women (*p* < .001). In the case of school years, those with higher education lived longer, indicating lower educational status as a risk factor for 5‐year mortality (*p* = .05). The diagnosis factor stayed the same, showing that AD patients had a higher risk of 5‐year mortality (*p* = .006). Lower TT3 levels still remained a significant risk factor (*p* = .004).

The fourth block included the GDS‐15 scores (representing depressive symptoms), which had significant impacts on the 5‐year mortality of patients (*p* = .009). Participants with higher GDS‐15 scores had a higher risk of dying. The fourth block also revealed the loss of significance for educational years (*p* = .056). All other parameters remained significant (*p* ≤ .009).

Finally, the fifth block incorporated the four factor‐score variables, representing neurocognitive abilities. Their impact on explaining the 5‐year mortality risk of the patients was substantial (*p* ≤ .006). The last model step considering all blocks together also revealed that cognitive diagnosis groups and school years no longer had a significant influence on patient 5‐year mortality risk in the present study (*p* > .05). Depressive symptoms, however, appeared to have had an impact on predicting the survival of patients (*p* = .004) but with low positive coefficients (*B* = 0.058) and a slightly elevated hazard ratio (HR) (1.060), meaning that the influence was rather small. Male gender (*p* < .001, reciprocal value of HR = 1.80) retained its significance in combination with the four neurocognitive factors that can be denoted as significant predictors of the 5‐year mortality of patients with neurodegeneration. Better neurocognitive functioning was revealed to be a significant (*p* ≤ .008) protection against 5‐year mortality (HR = 0.553 [*Verbal memory*] up to 0.821 [*Verbal fluency and semantic memory*]). Regarding all predictors together, there remained a tendency for lower TT3‐values to be associated with a higher risk of death, albeit the association did not have a significant influence on the 5‐year mortality of the patient collective. The prototypical TT3 difference between deceased and censored cases can be assumed as −0.58 ng mL^–1^; a lowered TT3 has a trend (reciprocal value of HR = 1.79) in predicting the 5‐year mortality of patients with cognitive degeneration, as shown in Table 3.

**TABLE 3:** Coefficients of Cox proportional hazards model, five blocks, containing all predictors, the final model (*n* = 931)

Predictor	*B*	SE	Wald (χ^2^)	*df*	*p* value	HR	95% CI for HR	
							LL	UL
TT3 (ng mL^–1^)	−0.581	0.330	3.096	1	.078°	0.560	0.293	1.068
Diagnosis			0.329	2	.848			
SCD vs. AD [1]	−0.192	0.365	0.279	1	.598	0.825	0.404	1.686
MCI vs. AD [2]	−0.030	0.258	0.023	1	.879	0.970	0.655	1.437
Gender (female)	−0.585	0.137	18.170	1	<.001**	0.557	0.426	0.729
School years	−0.014	0.019	0.558	1	.455	0.986	0.950	1.023
GSD‐15 score	0.058	0.020	8.365	1	.004**	1.060	1.019	1.102
NTBV *z*‐factor								
Attention and perceptual speed	−0.467	0.073	41.199	1	<.001**	0.627	0.544	0.723
Verbal memory	−0.592	0.086	47.657	1	<.001**	0.553	0.467	0.654
Executive functions	−0.206	0.064	10.257	1	.001**	0.814	0.717	0.923
Verbal fluency and semantic memory	−0.197	0.071	7.675	1	.006**	0.821	0.714	0.944

Abbreviations: CI, confidence interval; HR, hazard ratio; AD, Alzheimer's disease; MCI, mild cognitive impairment; SCD, subjective cognitive decline; TT3, triiodothyronine; GDS, Geriatric Depression Scale; NTBV, Neuropsychological Test Battery Vienna.

**p ≤ .01, *p ≤ .05, °*p* ≤ .10 (tendency).

## 4 DISCUSSION

Among the available thyroid parameters (TSH, TT3, fT3, T4, and fT4), only TT3 showed a difference in value between the MCI and AD patient populations. However, further analyses showed that that thyroid parameters, represented by TT3, had no significant influence on the survival and 5‐year mortality of the entire patient population with cognitive impairment (SCD, MCI, and AD), with a high reciprocal HR‐value. Our study did not show a connection between low TT3 values and increased 5‐year mortality risk among patients with cognitive impairment. Other studies showed a potential association between TT3 and the severity of cognitive degeneration,^23^ as well as the potential role of inhibiting β‐amyloid accumulation in the brain.^24^, ^25^ T3, free or total, is important because it suppresses the pathological process of neurodegeneration caused by dementia.^23^, ^24^, ^25^, ^26^ On the other hand, the decline of TT3 among AD patients compared to MCI patients might be one of many changes that comes with aging because the AD patient population is the oldest among the three diagnostic groups in the study. Jain^27^, when researching thyroid profiles, found that TSH, fT4, and TT4 levels increased, whereas fT3 and TT3 levels decreased with age in the healthy US population. This was further backed by a 2008 study reporting that, in healthy elderly individuals, T3 levels, free or total, decline with age.^28^ By contrast, Chiaravalloti et al.^29^ noted that AD‐affected individuals had significantly lower fT3 values than healthy individuals in the control group. Further data with more TT3, coupled with fT3 and fT4 values, could shed more light on the conclusions of the present study.

Gender showed a significant impact on the survival of cognitively impaired patients. It can be assumed that female patients tend to have a lower 5‐year mortality risk than their male counterparts. This has also been found in other studies.^30^, ^31^, ^32^, ^33^, ^34^ Education on the other hand, as represented in the study by school years, did not influence 5‐year mortality in the patient collective. Higher education is shown to be a protective factor,^35^ whereas another study from 1995 presented higher education with an increased mortality among patients with dementia as a result of a bigger cognitive ‘reservoir’, which the patient can potentially use to still appear mentally sound.^36^

Depressive symptoms, represented by GDS‐15, were significant predictors of 5‐year mortality among the patient collective, indicating that depressive symptoms can be seen as risk factors for mortality in patients with cognitive decline. However, the real effect is rather small. Depression in old age is associated with a two‐fold increase of both cardiovascular and non‐cardiovascular mortality in patients who are 85 years or older, even after adjustments for comorbidity conditions.^37^ Laudisio et al.^38^ found no increase in mortality in patients with depressive symptoms.

Cognitive performances represented by NTBV‐short was shown, as expected, to have an influence on the 5‐year mortality of patients with cognitive decline. Cognitive decline and neurocognitive functioning have been found to have an impact on mortality in other studies.^30^, ^39^ Thaler et al.^40^ also revealed that poor neuropsychological cognitive performance is correlated with a higher death toll, although it lost its significance after adjusting for comorbidities such as heart disease.

Although it did have a significant impact on 5‐year mortality until the last block of the Cox regression model, cognitive diagnosis lost this after taking NTVB factor scores into account. This is also substantiated by the fact that the age of death in the cognitive diagnosis groups is relatively the same regardless of the severity of the condition. This contrasts with several studies suggesting that cognitive decline has an impact on mortality.^2^, ^7^, ^9^, ^10^, ^41^, ^42^, ^43^, ^44^ This could be because diseases such as pneumonia, dehydration, and cachexia are more likely to occur in individuals dementia than in individuals from the healthy senior population.^9^, ^10^ One possible explanation is that social and psychological factors, rather than dementia itself, influence mortality.^10^ Furthermore, Mooldijk et al.^45^ showed that, in the general population, individuals with MCI die sooner than those without MCI, although the life expectancy is higher than in studies done in clinical settings. As mentioned above, the patient population with AD is significantly older than the rest of the study collective. Therefore, it is more deductive to attribute the mortality of the patients to age rather than cognitive impairment. Age and other variables, including gender, diabetes, cancer, and stroke, were considered as risk factors in mortality for patients with dementia in a longitudinal study in Taiwan.^34^ It should be noted that the present study compares the mortalities in individuals of a population already diagnosed with cognitive decline amongst each other and not with a healthy population, similar to that conducted in most previous studies reporting a coincidence between mortality and dementia.^2^, ^9^

The present study has several limitations. Although the study had 1511 participants, a large portion of the patient collective had incomplete datasets. In the final multi‐regression model, only 931 participants (61.2% of the whole patient collective) had all of the required data needed to be included in the regression model. One of the reasons why the thyroid data were incomplete was a result of the change in the laboratory results over the decades; TSH, TT3, and TT4 were regularly measured in the late 1990s up until the middle 2000s, whereas, after that point, thyroid examinations changed to TSH, fT3, and fT4. Second, the present study used multiple testing, which is susceptible to data‐dredging and bias‐finding of statistically significant results.^46^ To counter this bias, we used the Bonferroni adjustment. Finally, the present study investigated thyroid hormone levels in the serum and not in the cerebrospinal fluid.

In conclusion, the present study shows that TT3 has no significant impact on the survival and 5‐year mortality of patients afflicted with cognitive degeneration. To understand the relationship between thyroid parameters and dementia, further studies are needed. The data showed that female patients with cognitive impairment tend to live longer than male patients with cognitive impairment. The cognitive diagnosis did have an impact, until the last block of the Cox regression. Depressive symptoms appear to have had an impact on predicting the survival of patients, but the influence is rather small. Neurocognitive functioning is a valuable 5‐year mortality predictor in patients with cognitive impairment, and further exploration of their predictive value is recommended.

## CONFLICT OF INTERESTS

The authors declare that they have no conflicts of interest. A positive vote of the Medical University of Vienna from January 2020 with the EK‐Nr. 2040/2018 is available.

## AUTHOR CONTRIBUTIONS

**Blaž Đapić:** Data curation; Formal analysis; Investigation; Methodology; Project administration; Writing – original draft. **Eva Schernhammer:** Conceptualization; Data curation; Methodology; Supervision; Validation; Writing – original draft; Writing – review & editing. **Helmuth Haslacher:** Methodology; Supervision; Writing – review & editing. **Elisabeth Stögmann:** Conceptualization; Data curation; Methodology; Project administration; Supervision; Writing – review & editing. **Johann Lehrner:** Conceptualization; Data curation; Methodology; Project administration; Resources; Validation; Writing – review & editing.

## Data Availability

The data that support the findings of this study are available from the corresponding author upon reasonable request.
